# Smart Diagnostics: Combining Artificial Intelligence and In Vitro Diagnostics

**DOI:** 10.3390/s22176355

**Published:** 2022-08-24

**Authors:** Michael P. McRae, Kritika S. Rajsri, Timothy M. Alcorn, John T. McDevitt

**Affiliations:** 1 Department of Molecular Pathobiology, Division of Biomaterials, Bioengineering Institute, New York University College of Dentistry, 433 First Ave. Rm 822, New York, NY 10010, USA; 2 Department of Pathology, Vilcek Institute, New York University School of Medicine, 160 E 34th St, New York, NY 10016, USA; 3 Latham BioPharm Group, 6810 Deerpath Rd Suite 405, Elkridge, MD 21075, USA

**Keywords:** artificial intelligence, in vitro diagnostics, point of care, lab on a chip, immunoassay, cytology, clinical decision support tool

## Abstract

We are beginning a new era of *Smart Diagnostics*—integrated biosensors powered by recent innovations in embedded electronics, cloud computing, and artificial intelligence (AI). Universal and AI-based in vitro diagnostics (IVDs) have the potential to exponentially improve healthcare decision making in the coming years. This perspective covers current trends and challenges in translating Smart Diagnostics. We identify essential elements of Smart Diagnostics platforms through the lens of a clinically validated platform for digitizing biology and its ability to learn disease signatures. This platform for biochemical analyses uses a compact instrument to perform multiclass and multiplex measurements using fully integrated microfluidic cartridges compatible with the point of care. Image analysis *digitizes biology* by transforming fluorescence signals into inputs for learning disease/health signatures. The result is an intuitive *Score* reported to the patients and/or providers. This AI-linked universal diagnostic system has been validated through a series of large clinical studies and used to identify signatures for early disease detection and disease severity in several applications, including cardiovascular diseases, COVID-19, and oral cancer. The utility of this Smart Diagnostics platform may extend to multiple cell-based oncology tests via cross-reactive biomarkers spanning oral, colorectal, lung, bladder, esophageal, and cervical cancers, and is well-positioned to improve patient care, management, and outcomes through deployment of this resilient and scalable technology. Lastly, we provide a future perspective on the direction and trajectory of Smart Diagnostics and the transformative effects they will have on health care.

## 1. Introduction

A new era in health care is under way thanks to significant advances in clinical research, scalable chem- and biosensing technologies, embedded electronics, cloud-distributed software and services, and artificial intelligence (AI). Perhaps the one area of medicine most ripe for these innovations is in vitro diagnostics (IVDs). Roughly 70% of clinical decisions are directly influenced by diagnostic test results [1] which facilitate evidence-based patient care. However, the routine diagnostic testing modality, where samples are sent to a centralized testing laboratory, typically delays the availability of test results by days. This delay often limits the clinical utility of diagnostics and can increase patients’ anxiety as they wait for critical test results. Point-of-care (POC) diagnostics, such as those based on microfluidic and lab-on-a-chip (LOC) technologies, can deliver test results in minutes, thus enabling timely treatment decisions and minimizing patient anxiety.

A central goal of POC diagnostics is to enable new models of health care delivery where providers can achieve near-real-time diagnostic results, expediting treatment decisions and advice to patients. Despite these potential advantages, widespread transitions from lab-based testing to POC testing have been limited due to technological challenges, perceived higher costs, space limitations at clinical sites, and often poorer performance when compared with lab-based testing [2,3,4]. Another factor that has limited the adoption of POC technologies is the chronic staffing shortages in many clinical sites. In the United States, it is estimated that there will be a shortage of 124,000 physicians by 2033, and 200,000 nurses per year will need to be hired to meet increased demand and replace retiring nurses [5]. Consequently, there is a hesitance to increase the duties of clinic personnel with tasks such as testing. Therefore, there remains an unmet need for accurate POC tests that are minimally disruptive to the clinical workflow.

In the past decade, there has been extreme interest in AI, with medicine becoming the predominant industrial application of AI in terms of total equity funding [6]. The integration and implementation of AI in IVDs has the potential to revolutionize the status quo of assessing disease and health. Likewise, these AI-linked devices will outperform contemporary non-AI methods for diagnosing and prognosing diseases [7]. The FDA has recognized the value of AI to improve treatment decisions and has recently provided several guidance documents for manufacturers developing AI-driven clinical tools, including guidance for remote data acquisition, guidance for the development of clinical decision support tools, and guidance for the regulatory submission of stand-alone software-based medical devices [8]. Although significant challenges remain with integration and data acquisition [9], there are opportunities for universal and AI-based biosensor systems to usher in an era of *Smart Diagnostics*.

**Figure 1:**
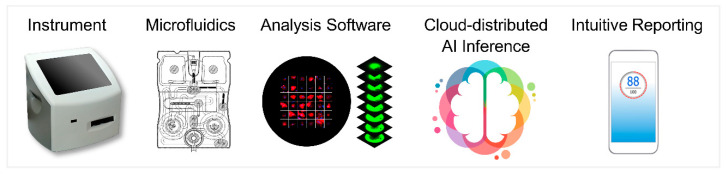
Elements of a Smart Diagnostics platform.

*Smart Diagnostics* are highly scalable IVDs which harness the power of AI to exceed the performance of lab-based diagnostics at a fraction of the cost. Furthermore, Smart Diagnostics are capable of deriving emergent properties through the novel detection and analysis of chemical and biological signatures and have the potential to exponentially improve health care in the coming years. Smart Diagnostics may have several key elements (Figure 1), including:

- A universal instrumentation platform that can facilitate a multitude of diagnostic tests;
- Highly scalable biosensors supported by microfluidics for assay processing;
- Analysis software for digitizing chemistry/biology;
- AI inference and clinical decision support;
- Intuitive reporting and integration with electronic health records.

Previously, we developed a flexible POC platform with integrated AI [10,11]. This Smart Diagnostics platform uses a compact instrument to process programmable assay cartridges—microfluidic devices pre-populated with liquid and dried reagents. Image analysis *digitizes biology* by converting fluorescence signals into features for machine learning algorithms trained to infer disease/health outcomes, resulting in an intuitive *Score* reported to patients and/or providers. This platform has been applied to predict outcomes in oral [12,13,14], ovarian [15], and prostate cancers, as well as cardiovascular disease [16], trauma, drug abuse [17,18], and COVID-19 severity [19,20]. This point-of-care compatible platform is capable of performing rapid immunoassays in about 10 min for drugs [17], 16 min for a COVID-19 severity panel [20], and 20–25 min for oral cytology [14]. The system completely automates the sample and reagent handling steps and thus requires minimal training to operate. Although the current benchtop device is intended for near-patient testing applications, the same technology is scalable to cheaper markets targeting resource-limited settings. To unlock the full potential of the Smart Diagnostics platform, disease/health scores can be monitored longitudinally to observe patient-specific changes over time to improve test sensitivity or to measure the treatment effect of drugs or therapies. Beyond testing, Smart Diagnostics could augment clinical care by linking to heterogeneous datasets (e.g., medical notes entered by physicians, medical images, continuous sensor monitoring, genomic analysis) [21]. This Perspective highlights key experiences in developing and validating Smart Diagnostics. The following sections summarize applications of Smart Diagnostics in cardiovascular disease, COVID-19, and cancer cytopathology.

## 2. Smart Diagnostics for Cardiovascular Disease

Cardiovascular disease (CVD) is the leading cause of death worldwide [22]. The costs attributed to CVD are a major burden on individuals and economies globally. There is a high potential to save lives and reduce the cost of care through the prevention, early detection, and management of CVD through AI-enabled clinical decision support tools which provide personalized assessments of health and disease based on diagnostic information [23].

Several groups have developed AI models for predicting CVDs. Artificial neural networks (ANNs) are commonly used for their excellent prediction performance and ability to learn complex nonlinearities in data, and they have been used for several published models, including: predicting acute myocardial infarction (AMI) in chest pain patients [24]; diagnosing AMI using cardiac enzyme data [25]; differential diagnoses of cardiac outcomes [26]; and discrimination of heart failure (HF) and chronic obstructive pulmonary disease (COPD) [27]. Alternate techniques have been used by other groups in the prediction of CVDs, including support vector machines [28], random forest [29], Bayesian networks [30], ensemble methods [31,32,33], and lasso logistic regression [34].

Towards the goals of reducing costs and saving lives, the McDevitt lab has developed the Cardiac ScoreCard—a universal multiplex cardiac biomarker assay paired with clinical decision support tools that provide both diagnostic and prognostic information across a spectrum of CVDs, including cardiac wellness, AMI, and heart failure (HF) [16]. The Cardiac ScoreCard assay comprises multiple cardiac biomarkers representing diverse CVD pathophysiology, a strategy which has been demonstrated to improve CVD risk predictions [35,36]. Additionally, this approach provides uncorrelated yet discriminatory predictors for training statistical learning models.

**Figure 2:**
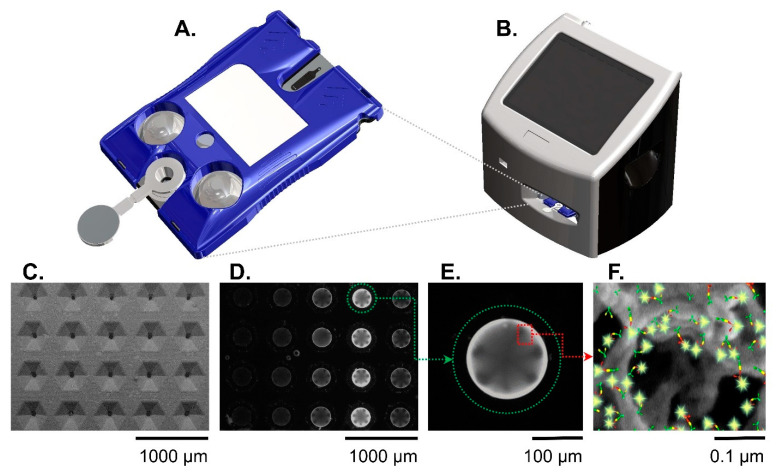
The Smart Immunoassay platform hardware consists of a cartridge (**A**) and a portable instrument (**B**). The instrument activates blister packs on the cartridge, performs the multistep immunoassay, and collects the immunofluorescent signal from the agarose beads. Panels (**C**–**F**) show the sensor(s) at different length scales. A scanning electron micrograph (**C**) shows the microfluidic cartridge’s sensor matrix without beads. A fluorescent image shows the same sensor matrix with beads present (**D**). Out of the 20 beads in the sensor matrix, a single agarose bead (encircled in green dotted line) is magnified (**E**) and shows a strong immunofluorescent reaction signal against a dark background. Panel (**F**) is a further magnified view of an agarose bead and illustration representing the fluorescent immunocomplexes formed on agarose bead fibers. The immunocomplexes are in sandwich configuration with capture antibodies (green symbols), antigen (yellow symbols), detecting antibodies (red symbols), and fluorophore (glowing yellow symbols). Reproduced from [20] with permission from the Royal Society of Chemistry.

The hardware for performing a Cardiac ScoreCard assay is shown in Figure 2. Using a small volume of serum (~100 μL), the cartridge performs an immunoassay, and the instrument converts the resulting fluorescence signal into biomarker concentrations. The single-use cartridges [11] are produced via injection-molding, and the molded fluidic body is sandwiched with laminate layers. The cartridge’s aluminum blisters, filled with phosphate-buffered saline, are compressed by actuators in the instrument to complete the immunoassay. The instrument contains a compact fluorescence microscope optimized for a high signal-to-noise ratio, and image analysis software converts the raw fluorescent signals to concentration measurements via a standard curve.

The Cardiac ScoreCard algorithms were developed for cardiac wellness testing and HF diagnosis applications. Detailed methods for model development and validation have been published previously [16]. Briefly, data from two clinical studies were merged to form training and test sets. The first study involved measuring serum cardiac biomarkers in 90 acute myocardial infarction (AMI) patients presenting to the emergency department and 100 recruited healthy controls [37,38]. The second study involved measuring cardiac biomarkers in patients presenting to the emergency department with chest pain or AMI-related symptoms (N = 389) [16]. The Cardiac ScoreCard algorithms implemented lasso logistic regression and considered 14 biomarkers (adiponectin, BNP, CD40L, creatine kinase-myocardial band [CK-MB], C-reactive protein [CRP], cardiac troponin I [cTnI], D-dimer, IL-1β, MMP-9, MPO, myoglobin [MYO], RANTES, sICAM-1, and TNF-α), and risk factors (age, gender, smoking, hypertension, and diabetes). The result is a single score interpreted as a probability of disease/wellness. The cardiac wellness model performed better than both the Framingham 10-year CVD risk score and a biomarker-only model in predicting high-risk patient groups measured in terms of area under the receiver operating characteristic curve (AUC) at 0.84, 0.80, and 0.77, respectively. The wellness model also demonstrated its utility as a continuous indicator for cardiac wellness with excellent calibration (Hosmer–Lemeshow *p* = 0.98). Similarly, the HF diagnosis model showed a slight improvement in discrimination compared with BNP alone with AUC = 0.94 and 0.93, respectively. In conclusion, this Cardiac ScoreCard approach demonstrates how Smart Diagnostics platforms can leverage shared strategic biomarkers across multiple clinical decision scenarios by training and validating new AI models—an efficient approach that significantly simplifies device development.

## 3. Smart Diagnostics for COVID-19

### 3.1. Predicting COVID-19 Severity in Patients with Cardiac Comorbidities

COVID-19 was first reported in Wuhan, China, in December 2019 [39,40] and declared a pandemic by the World Health Organization (WHO) on 11 March 2020 [41]. In response to the news of the global pandemic, the Cardiac ScoreCard assay [16] was quickly adapted to the task of predicting COVID-19 disease severity in patients with cardiac comorbidities. In less than three weeks, we had completed the training and initial validation of a COVID-19 disease severity model. By 11 April 2020, one month after the WHO declared a pandemic, we submitted our first publication featuring a clinical decision support tool that discriminates COVID-19 patients who recovered vs. those who died [20], representing the first scoring system for COVID-19 disease severity linked to POC biomarker tests.

Determining prognosis in high-risk individuals with COVID-19 was challenging throughout the pandemic. Early in the COVID-19 pandemic, evidence suggested that SARS-CoV-2 interacts with the cardiovascular system, and several studies linked COVID-19 prognosis to cardiac biomarkers [42,43,44,45,46], including cTnI, CRP, D-dimer, procalcitonin (PCT), N-terminal pro-B-type natriuretic peptide (NT-proBNP), and CK-MB. Patients suffering cardiovascular comorbidities experienced worse outcomes [47], and myocardial injury was higher in patients who died from COVID-19 [45,47].

**Figure 3:**
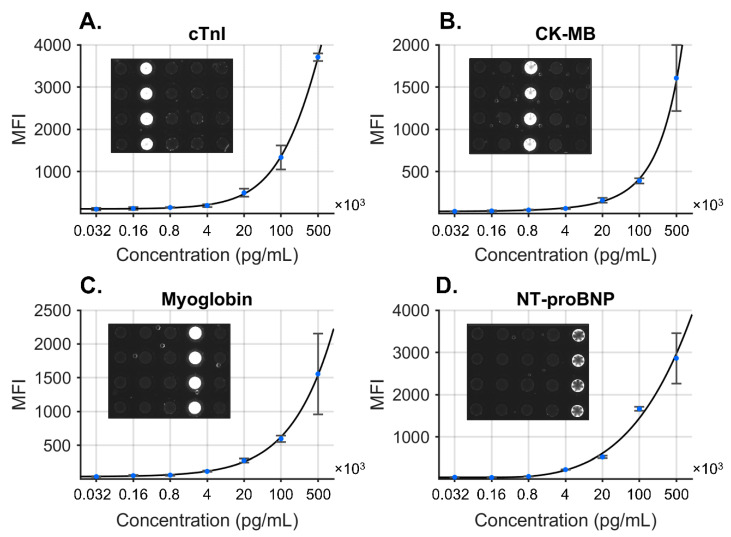
COVID-19 disease severity biomarker panel consisting of four biomarkers: cTnI (**A**), CK-MB (**B**), MYO (**C**), and NT-proBNP (**D**). Standard curves were fit to the concentration data, and specificity was demonstrated for each antigen at high concentration (inset images). Reproduced from [20] with permission from the Royal Society of Chemistry.

Leveraging our previous work developing the Cardiac ScoreCard, we efficiently adapted our Smart Diagnostics platform to the task of predicting COVID-19 severity in patients with cardiac comorbidities [20]. A direct sandwich immunoassay targeting cTnI, NT-proBNP, CK-MB, and MYO was developed using spherical agarose sensors functionalized with analyte-specific monoclonal antibodies for target capture. Secondary antibodies were conjugated to Alexa Fluor 488 for detection and deposited onto a glass fiber pad for elution within the cartridge. For each assay, twenty agarose microspheres were arranged with each column representing an analyte target. The assay sequence took approximately 15 min to complete the sample delivery, wash, detecting antibody delivery, and final wash. Images were acquired at the end of the run, and the signal from the outermost 10% of the microspheres was averaged. Standard curves were completed in triplicate to convert the mean fluorescence intensity (MFI) to concentration (Figure 3), and specificity was demonstrated using single antigen standards at high concentrations with minimal cross-reactivity.

After biomarker concentrations were measured, test values were transformed, along with clinically significant predictors, into prediction algorithms for the severity of COVID-19. A COVID-19 cardiac model was developed using data from COVID-19 patients presenting with hypertension. Out of the total 160 patients, 117 were discharged and 43 died. Biomarker values for PCT, CRP, MYO, CK-MB, and cTnI were significantly higher in the group that died versus those who were discharged. The COVID-19 Cardiac Score was trained to distinguish patients who recovered from those who died from complications, resulting in a model with the following predictors: age, sex, PCT, MYO, CRP, and cTnI. Median COVID-19 Cardiac Scores were significantly higher for those who died versus those who were discharged, resulting in an AUC (95% CI) of 0.94 (0.89–0.99). In conclusion, this study demonstrated strong potential for identifying COVID-19 patients with increased risk of mortality using a Smart Diagnostics approach and set the foundation for additional clinical decision support systems for COVID-19 prognosis.

### 3.2. Managing COVID-19 in a Community Health Network

The COVID-19 pandemic has caused significant morbidity and mortality, and the volume of patients requiring intensive care overwhelmed healthcare systems globally. Validated clinical decision support tools for COVID-19 can alleviate these problems by assisting in patient triage and resource allocation. Our previous work developed a model that specifically addressed COVID-19 prognosis in patients with hypertension; however, a more general tool was needed to assist in managing patients across the entire spectrum of risk for COVID-19 complications. Several predictive models for COVID-19 severity have been developed or adapted by other groups, including the Epic Deterioration Index [48], the Berlin Criteria for Acute Respiratory Distress Syndrome [49,50], the African Federation for Emergency Medicine [51], and the Brescia-COVID Respiratory Severity Scale [52]. However, none of these tools at the time were externally validated or had been developed specifically for COVID-19 patients.

We developed such a tool for managing COVID-19 patients which follows a tiered approach using easily obtainable non-laboratory inputs (Tier 1) and biomarkers typically measured in ambulatory settings (Tier 2). The typical workflow for the tool is as follows. Patients who test positive or are presumed positive for COVID-19 seek care at a community health clinic or emergency department where decisions are made in two stages. First, the Tier 1 Outpatient Score is calculated when laboratory data are not yet available and returns the probability of severe disease (ventilation or death) based on age, gender, systolic blood pressure, cardiovascular comorbidities, and diabetes status. Patients with Tier 1 Outpatient Scores below the low-risk threshold may be managed at home, whereas those with high scores are referred for a Tier 2 biomarker test. The Tier 2 Biomarker Score is the probability of mortality based on age, D-dimer, PCT, and CRP. Patients with a Tier 2 Biomarker Score below the low-risk threshold may be managed via Telehealth follow-up, whereas those with high scores may be hospitalized or managed with 24–48 h follow-up. Patients in hospital settings may also have their Tier 2 Biomarker Score monitored serially for evaluating disease progression or treatment effects.

The full details of the two-tier model development and validation have been described previously [19]. In summary, 701 patients with COVID-19 were seen across practices within the New York University (NYU) Langone Family Health Centers (FHC) network. Lasso logistic regression models discriminated patients who were not hospitalized or were discharged without ventilation, and patients who were ventilated or died. The Tier 1 model was externally validated with 160 hospitalized patients [45], and the Tier 2 model was externally validated with 375 hospitalized patients [53].

**Figure 4:**
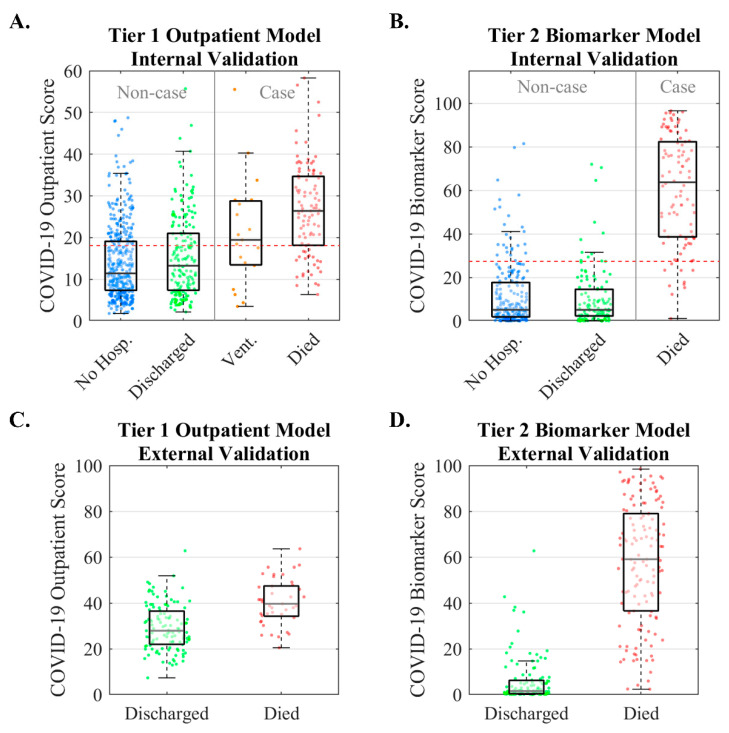
Internal and external validation results for the two-tiered COVID-19 disease severity models. The Tier 1 Outpatient Model is the probability of severe COVID-19 complications (ventilation or death) based on age, gender, systolic blood pressure, cardiovascular comorbidities, and diabetes status. The Tier 2 Biomarker Score is the probability of mortality from COVID-19 based on age, D-dimer, PCT, and CRP. Internal validation of the Tier 1 Outpatient Model (**A**) and Tier 2 Biomarker Model (**B**). External validation for Tier 1 Outpatient Model (**C**) and Tier 2 Biomarker Model (**D**). (No Hosp. = patients who were not hospitalized, Vent. = patients who were ventilated). Reproduced from [19] under the terms of Creative Commons Attribution 4.0 license.

Internal and external validation results for the Tier 1 and Tier 2 models are summarized in Figure 4. Median COVID-19 Outpatient Scores increased across patient groups (11, 13, 20, and 27 for not hospitalized, discharged, ventilated, and deceased patients, respectively). The model’s AUC (95% CI) was 0.79 (0.74–0.84). The Tier 2 Biomarker Score is the probability of mortality based on age, D-dimer, PCT, and CRP. Median COVID-19 Outpatient Scores were 5, 5, and 64 for not hospitalized, discharged, and deceased patients, respectively (statistically significant differences for comparisons between not hospitalized vs. died [*p* < 0.001] and discharged vs. died [*p* < 0.001]). The model’s AUC (95% CI) was 0.95 (0.92–0.98). External validation of the Tier 1 Outpatient Model evaluated 160 hospitalized COVID-19 patients who were either discharged or died [45]. COVID-19 Biomarker Scores were lower in patients who were discharged than those who died, with an AUC of 0.79 (0.70–0.88). External validation of the Tier 2 Biomarker Model evaluated 375 hospitalized COVID-19 patients who were either discharged or died [53]. The median (interquartile range) COVID-19 Biomarker Scores were 1.6 (0.5–6.2) for patients who were discharged and 59.1 (36.6–78.9) for patients who died, with an AUC of 0.97 (0.95–0.99).

Showing generalization through external validation, as demonstrated for the two-tier COVID-19 severity models, is an essential step for translating prediction models to clinical practice [54]. Shortly after publishing the models, the COVID-19 Biomarker Score was further evaluated for predicting survival to discharge in patients evaluated for percutaneous dilational tracheostomy [55], where the novel score found utility in determining which infected patients would benefit from tracheostomy. Another strength of this approach is its interpretability. Many AI algorithms are “black boxes” (i.e., their decision rationale is uninterpretable), although the lasso logistic regression approach used in both the Cardiac ScoreCard and COVID-19 scores is fully transparent and easy for clinicians to interpret. This Smart Immunoassay platform in addition to transparent clinical decision support tools is ready to assist healthcare providers in making evidence-based decisions in managing cardiac and COVID-19 care with a strong potential for improving patient outcomes and reducing costs.

## 4. Smart Diagnostics for Cancer Cytopathology

### 4.1. Oral Cancer Cytopathology

Oral potentially malignant disorders (OPMDs) are oral lesions which require additional testing to diagnose oral epithelial dysplasia (OED) or oral squamous cell carcinoma (OSCC). The gold standard for diagnosing OPMDs involves specialist referral, invasive scalpel biopsy, and histopathological evaluation. Oral cancer survival substantially improves when the disease is detected in its early stages; however, many malignant and pre-malignant lesions are identified late due to inadequate screening. It is challenging for doctors and dentists to make referral decisions based on a visual inspection and risk factors alone [56].

Numerous screening tools that are less invasive than scalpel biopsy and histopathology are available for assisting dentists in OPMD triage. For example, light-based adjuncts such as VELscope, ViziLite Plus, and Microlux DL allow physicians to screen suspicious lesions at the POC; however, their diagnostic utility as screening adjuncts remains unproven [57,58]. Despite the availability of these screening tools, only cytology is considered suitable as a surrogate for histopathology [56,57,58]. There are several commercial cytology lab services available, including OralCDx BrushTest [59], Forward Science CytID [60], and Resolution Biomedical Clear Prep [61]. Despite the availability of commercial cytology services, delays associated with remote testing and biased validation studies [62,63] have dampened enthusiasm for cytology adjuncts. There is a sustained need for a Smart Cytology platform with the sensitivity to discriminate lesions of clinical significance.

We previously described a cytology-on-a-chip platform for oral cancer screening comprising microfluidic flowthrough devices, multispectral imaging, and analysis of single cells [64]. This approach was validated in a large clinical study in which the cytology-on-a-chip measurements were associated with six levels of histopathological diagnoses [12,65]. More recently, this lab-based cytology approach was translated to a POC Smart Cytology platform comprising a brush cytology sampling kit, single-use assay cartridge, portable instrument, improved clinical algorithms, and automated AI analysis capable of learning and identifying cytological signatures predictive of OED and OSCC from thousands of single cells in a matter of minutes [14]. Using this Smart Cytology approach, we discovered a novel cell phenotype—differentiated squamous epithelial cells with nuclear F-actin (i.e., cells with F-actin in or around the nucleus) [13]. This nuclear F-actin signature was the single best predictor for discriminating severe dysplasia out of 188 other predictors from cytology and represents the first study of its kind to elucidate nuclear F-actin in predicting early OED. The following sections summarize the development, discovery, and validation of this Smart Cytology platform.

### 4.2. Training and Validation Data

The Smart Cytology platform was evaluated in a cross-sectional study of 999 subjects recruited prospectively [12,65]. Significantly, this effort identified an expanded group of promising biomarkers (EGFR, β-catenin, Geminin, αvβ6, CD147, McM2, and Ki67) for use in the classification of mucosal lesions across six classes of histopathologic diagnoses, with the primary objective of distinguishing between benign, dysplastic, and malignant lesions. Cytology measurements from 714 subjects were completed with >200 cellular features related to biomarker expression and nuclear/cellular morphology per cell, representing ~2000 cells per patient, or roughly 13 million indexed cell objects.

Brush cytology specimens and matched scalpel biopsies from 486 subjects from three groups were included in the analysis: (1) subjects with OPMD, (2) previously diagnosed malignant lesions, (3) and healthy volunteers without lesions. Histopathological assessment classified scalpel biopsies into six categories based on the WHO guidelines [66] using a multi-stage adjudication process [65] which overcomes limitations of conventional OED grading [67].

### 4.3. Machine-Learning-Based Cell Phenotype Classifier

**Figure 5:**
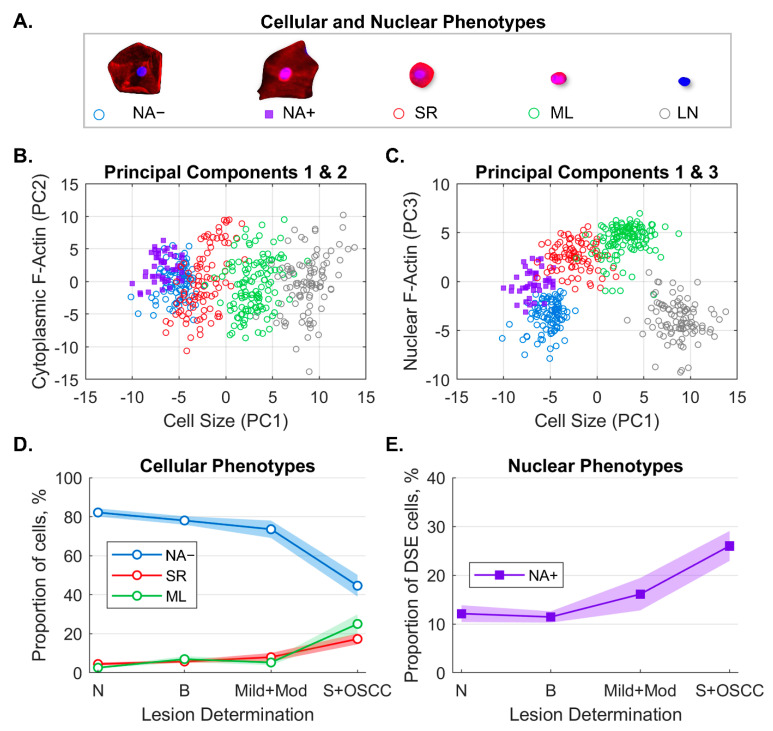
Machine learning algorithm to classify and count cellular and nuclear phenotypes. Five cellular/nuclear phenotypes were identified (**A**). Principal component analysis of phenotypes shows clusters of phenotypes for PC1 vs. PC2 (**B**) and PC1 vs. PC3 (**C**). The majority of variance was explained by cell size (PC1), cytoplasm F-actin (PC2), and nuclear F-actin (PC3). Distributions of cellular phenotypes (**D**) and nuclear phenotypes (**E**) identified by machine learning within each lesion class (solid line = cell percentages, fill = 95% CI). Panel E shows the fraction of NA+ cells out of all DSE cells. NA− = differentiated squamous cells without nuclear F-actin; NA+ = differentiated squamous cells with nuclear F-actin; SR = small round cells; ML = mononuclear leukocytes; LN = lone nuclei; PC = principal component; DSE = differentiated squamous epithelial cells; N = normal lesion (n = 121); B = benign lesion (n = 241); Mild+Mod = mild and moderate dysplasia (n = 50); S+OSCC = severe and oral squamous cell carcinoma (n = 74). Reproduced from [13] with permission from SAGE Publishing.

Machine learning classifiers (*k*-nearest neighbor) were trained to identify and count cell phenotypes predictive of OED and OSCC (Figure 5), including the following:

- Immature basaloid keratinocytes or small round (SR) cells appearing as small, circular cells 12–30 µm in diameter;
- Mononuclear leukocytes (MLs) appearing as brightly stained pink cells 6–23 µm in diameter;
- Lone nuclei (LN) appearing as cell objects 5–12 µm in diameter with DAPI counterstaining but no cytoplasmic F-actin staining;
- Differentiated squamous epithelial cells (DSE) or mature keratinocytes appearing as broad/flat cells 50–100 µm in diameter;
  ○ DSE cells with nuclear actin (NA+);
  ○ DSE cells without nuclear actin (NA−).

Principal component analysis (PCA) of the cell phenotype data showed that the majority of variance was explained by the first three principal components which were labelled as latent variables: cell size (33%), cytoplasmic F-actin (14%), and nuclear F-actin (14%). This result suggested that cell size and F-actin distribution within the cell play the largest role in differentiated cell phenotypes. Furthermore, cell phenotypes were demonstrated to correlate well with more advanced disease, with the proportion of NA+ cells increasing with disease severity (Wilcoxon rank sum *p* < 0.05 for all OED and OSCC).

### 4.4. Predicting a Spectrum of OED/OSCC

Predictive models were developed using cytology data (percentages for cell phenotypes), lesion characteristics (lesion area, color [red, white, or red and white], and clinical appearance of lichen planus), and risk factors (sex, age, and smoking). Models were trained to discriminate the histopathology grade across multiple dichotomous splits (Table 1). Diagnostic accuracy was measured in terms of the AUC, sensitivity, and specificity for each split, including an *early disease* model (benign vs. all lesions of greater severity) and a *late disease* model (lesions of low and moderate severity vs. all lesions of greater severity).

As expected, the late disease model was more accurate than the early disease model, with AUCs of 0.93 and 0.82, respectively. Higher percentages of SR and ML were associated with late disease only, confirming prior studies that observed higher frequencies of SR cells and ML in high-grade OED and OSCC [68,69]. Interestingly, higher proportions of NA− cells were associated with lower odds of OED and OSCC, whereas the proportions of NA+ cells were positively associated with both early and late disease. We hypothesized that DSE cells with nuclear F-actin could be transitioning in morphology from the NA+ to SR. Importantly, this study was the first to associate nuclear F-actin cells with an increased risk of OED. These results demonstrated that features from cytology could substantially improve predictions of OSCC over models relying solely on risk factors and lesion appearance. This points to the strong potential for AI-based cytology to improve the screening and surveillance of the entire spectrum of OPMD in multiple care settings. These settings include primary care and dental care applications, to differentiate the significance of common oral mucosal lesions; and in secondary or tertiary care settings, to longitudinally monitor patients with a history of OED and OSCC and identify lesions with potential for progression of dysplasia, malignant transformation, or cancer recurrence.

## 5. Discussion and Outlook

### 5.1. Considerations for Detection Methods

There are a variety of detection techniques for visualization following immunodetection, such as colorimetric, fluorescence, chemiluminescence, bioluminescence, chemifluorescence, autoradiographic, and immunogold labelling [70]. In general, any of these methods would be suitable for Smart Diagnostics; however, fluorescence detection has several advantages. Compared with colorimetric detection, fluorescence has improved sensitivity and a wider dynamic range by enabling the detection of higher concentrations without sample dilution [71,72,73,74,75]. Relative to chemiluminescence methods, they offer a tenfold wider dynamic range and improved linearity [76]. Moreover, the use of multiple fluorophores with unique excitation/emission spectra allows multiplexing targets within the same spatial region—an attractive attribute for cell imaging applications. Lastly, fluorescent markers are relatively stable for long periods of time under the right conditions. Despite these advantages, there are a few limitations including light scattering, background fluorescence, autofluorescence, nonspecific sources of noise (e.g., dust, debris, and bubbles), and photobleaching. Significant efforts have been dedicated to minimizing these sources of variability in the system in order to achieve laboratory-quality results in integrated systems.

### 5.2. Considerations for AI Methods

There is no one-size-fits-all approach to selecting and developing AI or machine learning models for integration in IVDs. The challenge of developing AI for IVDs is complex, requiring expertise across multiple disciplines involving not just extensive statistical and machine learning expertise, but also domain knowledge of clinical applications and an understanding of the development of regulated IVDs. There are many considerations when developing AI or machine learning algorithms for integration with IVDs. Such factors include (1) the learning model (i.e., supervised, unsupervised, or reinforcement learning); (2) the learning task (classification, regression, prediction, clustering, or dimensionality reduction); (3) the size of the dataset available for training, testing, and validation (i.e., the number of samples [rows] and predictors [columns]); (4) the type and structure of the data (numerical [continuous or discrete], categorical, ordinal, cross-sectional in time vs. time-series, etc.); and (5) the interpretation of algorithm results (i.e., black box response or analysis requiring interpretation). Domain knowledge of the problem can also influence model selection and development. For example, problems with a few established risk factors may perform satisfactorily with simpler approaches and fewer covariates (e.g., logistic regression, naïve Bayes, and decision trees). More challenging problems, especially those with a high ratio of predictors to samples, may require exploratory analysis with dimension reduction (principal component analysis [PCA], independent component analysis [ICA], and t-distributed stochastic neighbor embedding [t-SNE]) and shrinkage/selection to improve interpretation and generalization (e.g., ridge, lasso, and elastic net). Additionally, domain knowledge of the problem can inform whether simple linear or advanced nonlinear methods are required. The level at which AI is integrated with the diagnostics also influences the selection of the algorithm. Here, algorithm selection will vary depending on whether it is applied to hardware/sensor data, the analysis of images versus tabulated data, or the prediction of clinical outcomes from data compiled from multiple disparate sources and/or mixed data structures. Given the diversity and complexity of challenges faced by AI developers and the rich set of tools available, the future of Smart Diagnostics will embody a heterogeneous collection of AI algorithms and architectures that are fine-tuned to meet the specific needs of the problems being addressed.

### 5.3. The Future of Smart Cytology

The future of Smart Cytology will likely involve deep learning—a relatively new field of AI enabled by multilayered neural networks learning from vast amounts of data. For cytology applications, one of the major advantages of deep learning over conventional machine learning is feature extraction. Machine-learning-based cytology involves extracting numerical data from morphological and intensity-based information from images of cells. The resulting tabulated data are then used to train algorithms to classify and count cell phenotypes. Although machine learning approaches are compatible with smaller datasets, they require extensive domain knowledge to curate a set of features from cytology with clinical significance. On the other hand, deep learning does not require a feature extraction step (i.e., the network learns directly from the raw data). However, large neural networks require massive amounts of data to improve performance relative to traditional machine learning methods. Traditional machine learning algorithms plateau in performance with additional data, whereas deep learning model performance tends to increase as the dataset size increases. These advantages make deep-learning-based Smart Cytology platforms increasingly attractive for detecting cytological signatures that are otherwise intractable to identify with simpler machine learning approaches. For this area, we are currently evaluating our Smart Cytology platform in a prospective longitudinal study of the malignant transformation of OED and recurrence of OSCC in which we develop deep learning models to detect rare cell phenotypes of malignant transformation or cancer recurrence, including nuclear actin signatures, migratory cell phenotypes, cell attachments, stress fibers, cell cycle signatures, and F-actin foci. We hypothesize that the identification of these molecular-level phenotypic changes, assisted by deep learning, will precede visual macroscopic changes in the lesion, thus providing a novel method for the earlier detection of malignant transformations or OSCC recurrence.

Future applications of Smart Cytology may target additional oral mucosal diseases (e.g., immune-based diseases such as lichen planus or pathogen-mediated diseases such as candida leukoplakia, often classified as differential diagnosis to OSCC) as well as other carcinomas such as lung, colorectal, esophageal, bladder, and cervical cancers. In general, changes to cellular actin have been implicated in cancer initiation and progression [77,78], in which increased cell motility governed by actin–myosin contraction, cell adhesion, and actin polymerization helps cancer cells invade, spread, and grow [79]. In the nucleus, actin serves a variety of functions such as organizing the nucleus [80], mechanosensing [81], nuclear expansion [82], and increasing nuclear compliance while protecting genetic material [83]. Furthermore, the use of nuclear actin as a cross-indication biomarker for bladder cancer risk has been studied in vitro in uroepithelial cell lines [84]. Given the numerous roles and ubiquity of cellular and nuclear actin in cancer cells, a Smart Cytology platform capable of recognizing and quantifying these cytological signatures will have clinical significance across multiple cancers. Likewise, the ability to perform multiple fluorescent counterstains and multispectral imaging creates unique opportunities to add future biomarker targets that may extend the platform’s flexibility and utility. Although the translational diagnostic utility of cytological signatures has already been demonstrated for OED and OSCC, future clinical validation studies are needed to validate the platform for additional cancer cytopathology applications.

### 5.4. Smart Diagnostics for Longitudinal Monitoring

One of the most exciting features of Smart Diagnostics is in the area of personalized medicine and the ability to monitor and learn patterns from individuals over time. This AI-guided *precision diagnostic* approach attempts to detect early signs of disease at the individual level rather than applying global cutoffs at the population level. Such precision diagnostic approaches can address the inherent biological (within-individual) variability associated with diagnostic measurements to make better predictions of disease/health status in individuals with different baseline conditions. For example, in a study of longitudinal measurements of CA-125 for the prediction of ovarian cancer, the applications of personalized thresholds for biomarkers would have captured all but one case of ovarian cancer at the same time or earlier than the population thresholds [85]. Interestingly, the personalized thresholds would have detected ovarian cancer about one year earlier on average relative to the population thresholds. This precision diagnostic approach has significant implications for the field of in vitro diagnostics and will play a prominent role in the future of Smart Diagnostics.

Conventional clinical studies collect a limited number of measurements from thousands of subjects to investigate the characteristics of a population or subgroups (e.g., drug treatment trials to identify responders versus non-responders). However, personalized medicine requires a different type of clinical study in which single-person studies, or *N*-of-1 trials, are needed [86,87]. Here, a larger number of measurements are collected on subjects over a clinically significant period of time, and aggregate results from these subjects can facilitate the development of more sensitive diagnostics. Such a study is now being conducted by our team in the area of monitoring patients with known OED for malignant transformation and patients with a history of OSCC for cancer recurrence. Through this study, we hope to usher in a new paradigm of *precision lesion diagnostics*, in which each patient may be monitored for their own risk profile with the goal of providing an earlier and more accurate lesion diagnosis that will improve patient survival and quality of life. In the future, improvements in Smart Diagnostics technology will make these devices cheaper, less invasive, and more accessible. As a result, the use of personalized thresholds, rather than population thresholds, will become the norm in health monitoring applications.

### 5.5. Future of Smart Diagnostics

We are now at the beginning of the AI era, and the expectations for AI are high in several specialties in medicine, including radiology, oncology, and general clinical decision-making. AI is beginning to have an impact for clinicians, in the form of rapid and accurate interpretation of imaging; for health systems, by improving workflow and reducing errors; and for patients, by empowering them with their own health data [88]. As of 2020, there have been 64 AI-based devices and algorithms cleared or approved by the FDA, of which 85.9% received FDA 510(k) clearance, 12.5% received de novo clearance, and 1.6% received premarket approval (PMA) [89]. However, despite the enormous potential of AI in medicine, the translation of these tools is hindered by several challenges including issues with transparency, bias in algorithm training and validation, and issues with privacy and security. In the next 5–10 years, Smart Diagnostics will not involve some broad “all-knowing” AI with generalized intelligence (e.g., IBM Watson), but rather narrow algorithms trained by carefully curated datasets specifically targeted to their indications for use. Such universal and AI-based IVDs have the potential to exponentially improve health care in the coming years.

## Figures and Tables

**Table 1:** Diagnostic accuracy of predictive models for OED/OSCC. Dichotomous splits for case vs. non-case are indicated by “|”. Sensitivity, specificity, and AUC (95% CIs) for the cross-validated algorithms for early disease (2|3,4,5,6), mild|moderate dysplasia (2,3|4,5,6), low|high risk (2,3,4L|4H,5,6), late disease (2,3,4|5,6), benign vs. malignant (2 vs. 6), and healthy control vs. malignant (1 vs. 6) models. Reproduced from [13] with permission from SAGE Publishing.

	Sensitivity	Specificity	AUC
Early Disease—2|3,4,5,6	0.72 (0.67–0.76)	0.73 (0.69–0.78)	0.82 (0.77–0.87)
2,3|4,5,6	0.79 (0.74–0.83)	0.85 (0.81–0.89)	0.89 (0.84–0.93)
2,3,4L|4H,5,6	0.80 (0.75–0.84)	0.82 (0.78–0.86)	0.89 (0.84–0.93)
Late Disease—2,3,4|5,6	0.86 (0.82–0.90)	0.84 (0.80–0.88)	0.93 (0.88–0.97)
2 vs. 6	0.89 (0.85–0.92)	0.90 (0.85–0.93)	0.95 (0.91–0.98)
1 vs. 6	0.94 (0.89–0.97)	0.92 (0.87–0.95)	0.97 (0.94–1.00)
